# Relevance of biomarkers indicating gut damage and microbial translocation in people living with HIV

**DOI:** 10.3389/fimmu.2023.1173956

**Published:** 2023-04-21

**Authors:** Jing Ouyang, Jiangyu Yan, Xin Zhou, Stéphane Isnard, Vijay Harypursat, Hongjuan Cui, Jean-Pierre Routy, Yaokai Chen

**Affiliations:** ^1^ Department of Infectious Diseases, Chongqing Public Health Medical Center, Chongqing, China; ^2^ Clinical Research Center, Chongqing Public Health Medical Center, Chongqing, China; ^3^ Cancer Center, Medical Research Institute, Southwest University, Chongqing, China; ^4^ Infectious Diseases and Immunity in Global Health Program, Research Institute, McGill University Health Centre, Montréal, QC, Canada; ^5^ Chronic Viral Illness Service, McGill University Health Centre, Montréal, QC, Canada; ^6^ Canadian HIV Trials Network, Canadian Institutes for Health Research, Vancouver, BC, Canada; ^7^ Division of Hematology, McGill University Health Centre, Montréal, QC, Canada

**Keywords:** HIV infection, biomarker, intestine, microbial translocation, inflammation

## Abstract

The intestinal barrier has the daunting task of allowing nutrient absorption while limiting the entry of microbial products into the systemic circulation. HIV infection disrupts the intestinal barrier and increases intestinal permeability, leading to microbial product translocation. Convergent evidence has shown that gut damage and an enhanced level of microbial translocation contribute to the enhanced immune activation, the risk of non-AIDS comorbidity, and mortality in people living with HIV (PLWH). Gut biopsy procedures are invasive, and are not appropriate or feasible in large populations, even though they are the gold standard for intestinal barrier investigation. Thus, validated biomarkers that measure the degree of intestinal barrier damage and microbial translocation are needed in PLWH. Hematological biomarkers represent an objective indication of specific medical conditions and/or their severity, and should be able to be measured accurately and reproducibly *via* easily available and standardized blood tests. Several plasma biomarkers of intestinal damage, i.e., intestinal fatty acid-binding protein (I-FABP), zonulin, and regenerating islet-derived protein-3α (REG3α), and biomarkers of microbial translocation, such as lipopolysaccharide (LPS) and (1,3)-β-D-Glucan (BDG) have been used as markers of risk for developing non-AIDS comorbidities in cross sectional analyses and clinical trials, including those aiming at repair of gut damage. In this review, we critically discuss the value of different biomarkers for the estimation of gut permeability levels, paving the way towards developing validated diagnostic and therapeutic strategies to repair gut epithelial damage and to improve overall disease outcomes in PLWH.

## Introduction

The introduction and extensive usage of antiretroviral therapy (ART) for HIV infection has resulted in persistent inhibition of viral replication, and a dramatic decline in morbidity and mortality in people living with HIV (PLWH). However, ART does not comprehensively restore the compromised immune system, and a chronic state of inflammation persists in PLWH on ART, even after long-term viral suppression. This chronic inflammation is positively associated with non-AIDS comorbidities and premature aging (1–5).

The gut epithelial barrier acts as an essential player in maintaining intestinal homeostasis, and in restricting the entry of microbes and their pro-inflammatory products through the mucosa and into the systemic circulation (6–12). The human gut is inhabited by a microbiota population comprising nearly 100 trillion individual organisms (bacteria, archaea, fungi, and viruses) (13, 14). Host-microbial mutualism in the intestine contributes to intestinal homeostasis (11, 13, 15). However, the gut is one of the earliest targets of HIV, as the virus is known to induce dramatic alterations to the gut microbiota and the gut mucosa (16–19). Epithelial damage allows microbial products to translocate from the gut lumen into the systemic circulation, to subsequently participate in and contribute to the chronic inflammatory state present in PLWH (2, 20–22).

A direct method to determine gut integrity is *via* intestinal biopsy during endoscopy; however, this is relatively invasive and is not suitable or appropriate in large populations (23). Another method to measure gut integrity is to determine the urinary excretion of a sugar probe or other labeled molecule that is not usually absorbed by the intestine [such as the lactulose-mannitol test (24) and the 51Cr-EDTA test (25)], which indirectly reflects intestinal permeability. However, these tests are time-consuming, lack standardization, and have relatively limited validity (24, 26).

Plasma or serum biomarkers can easily be identified from blood samples and are considered to be non-invasive tests for the accurate diagnosis and prognosis of disease. Several gut damage biomarkers, including intestinal fatty acid-binding protein (I-FABP), zonulin, and regenerating islet-derived protein-3α (REG3α) have been validated as biomarkers of gut damage, as well as markers of microbial translocation such as lipopolysaccharide (LPS), LPS-binding protein (LBP), sCD14, (1, 3) β-D-Glucan (BDG). These markers are mainly used in studies related to colonic inflammation (and only more recently in HIV studies), where each marker may have differing values for diagnosis and prognosis (27–32). Herein, we summarize published information regarding these gut damage and microbial translocation biomarkers in PLWH, and also discuss potential therapeutic strategies to potentially improve the integrity of the intestinal barrier assessed using these biomarkers.

## Key factors associated with intestinal damage in PLWH

**Figure 1:**
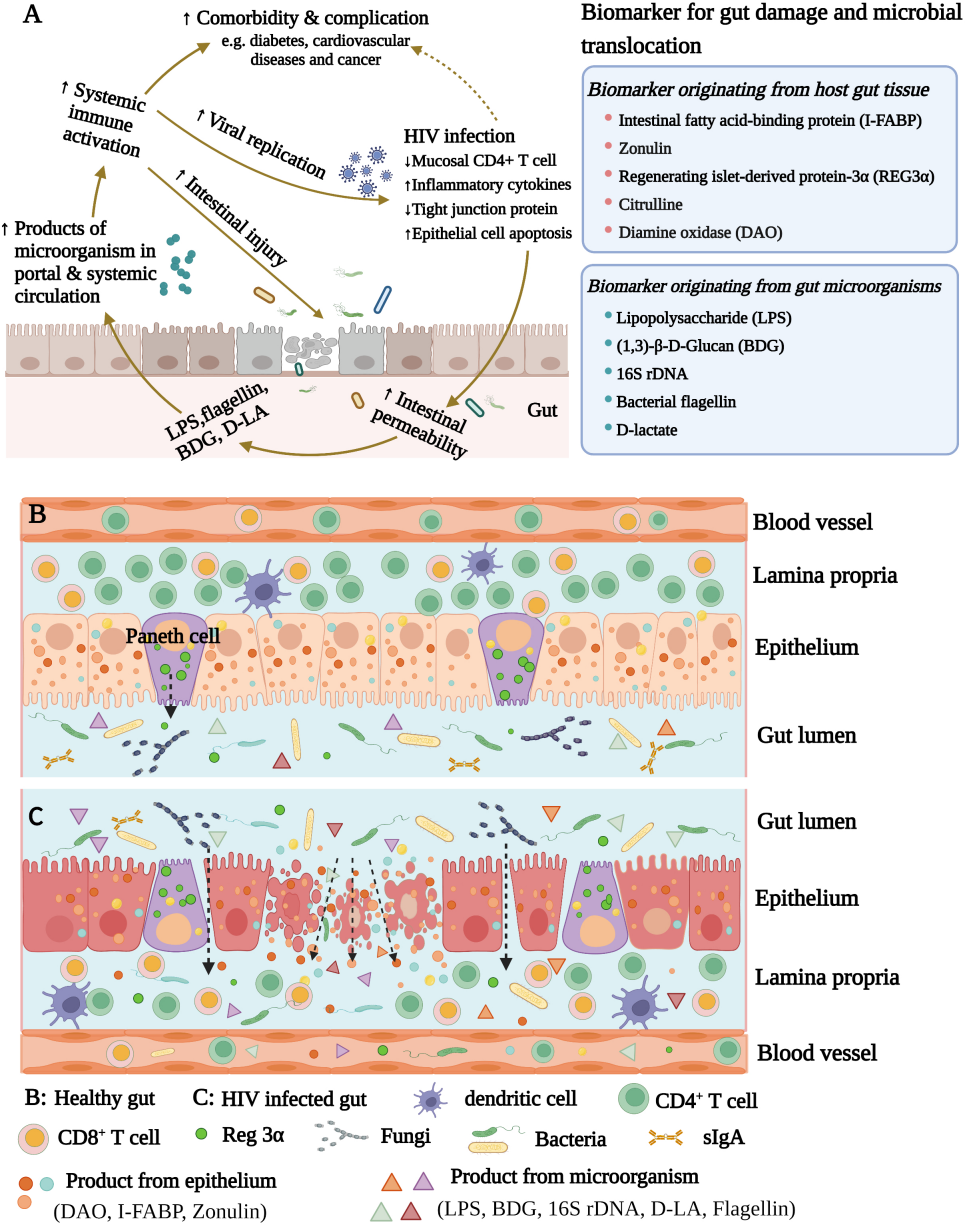
**(A)** The microbial translocation caused by HIV infection increases systemic inflammation. HIV infection damages the intestinal barrier and increases intestinal permeability. The intestinal microorganisms and their products subsequently enter into blood circulation, inducing immune activation and increasing the systemic inflammatory response. **(B)** Healthy gut. **(C)** HIV infected gut. HIV infection damages the intestinal barrier and promotes intestinal permeability.

**Figure 2:**
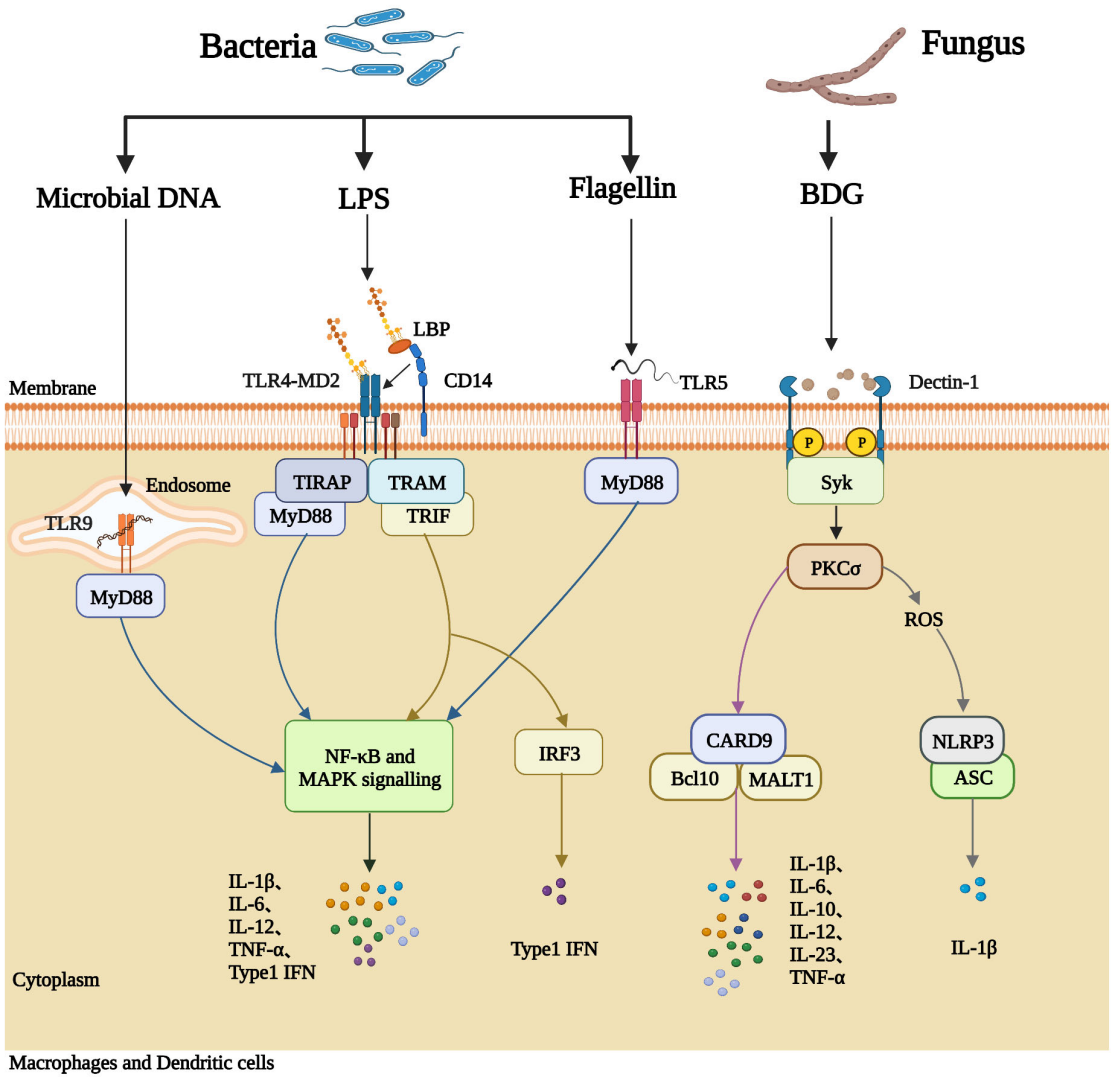
Microbial products induce immune activation in PLWH. Macrophages and dendritic cells express pattern recognition receptors (PRRs) on the cell membrane, which can recognize pathogen-associated molecular patterns (PAMPs) on the surface of microorganisms, including bacterial DNA, LPS, flagellin, and fungal BDG, and subsequently generate inflammatory responses. LPS-binding protein (LBP) and CD14 deliver LPS to the signaling receptor complex TLR4/MD2 on the outer member of the cell. The TLR4/MD2/LPS complex activates the NF-κB signaling pathway and the mitogen-activated protein kinase (MAPK) signaling pathway, causing the synthesis and release of pro-inflammatory cytokines into the blood (such as IL-6, IL-12, TNF-α, and IL- 1β). Membrane TLR5 recognizes bacterial flagellin and activates NF-κB through MyD88. Bacterial DNA can be recognized by TLR9 located in the endosome, activating the MyD88-dependent pathway. BDG from fungi is recognized by the C-type lectin receptor Dectin-1 and mediates inflammatory cytokine production by activating Syk-dependent pathways. The downstream signal PKCσ from Syk (complex phosphatase SHP2) leads to the production of reactive oxygen species (ROS) and activates CARD9/Bcl-10/Malt-1 complex, causing the synthesis and release of pro-inflammatory cytokines into the blood (such as IL-6, IL-10, IL-12).

Intestinal structural and immunological damage is common in PLWH, leading to increased gut permeability, microbial translocation, and subsequent immune activation (33–36). An overview of this process is summarized in **Figure 1**, and the mechanisms whereby immunological activation is induced by some microbial products are illustrated in **Figure 2**. Cumulative evidence has shown that during HIV infection, the processes of intestinal crypt hyperproliferation and villous shortening results in partial villous atrophy during all stages of HIV infection (37–40). Li et al., reported that in rhesus macaques, simian immunodeficiency virus (SIV) infection induces massive apoptosis of intestinal epithelial cells, and this apoptosis is directly related to the depletion of gut lamina propria CD4^+^ T-cells (41). A study by Epple et al., using immunofluorescence visualized increased apoptosis of duodenal epithelial cells in patients with acute and chronic HIV infection (42). Another intestinal permeability study showed higher lactulose-mannitol ratios in HIV-infected patients compared to uninfected control individuals, indicating that gut permeability is increased in PLWH (43). Moreover, various other gut microbial products, such as LPS, 16S rDNA, and BDG, have now been identified in the circulation of PLWH (2, 21, 29, 44).

Several factors contribute to the gut damage seen in PLWH. Firstly, HIV may directly induce epithelial apoptosis and thus directly damage the gut epithelial barrier (45–47). *In vitro*, exposure to HIV-1 gp120 (a surface envelope glycoprotein) impairs mucosal epithelial barrier integrity by reduction of tight junction (TJ) proteins, resulting in increased epithelial permeability and microbial translocation (46). Other reports have observed that the HIV-1 Tat protein (an HIV regulatory protein) may inhibit proliferation of, and promote apoptosis in intestinal epithelial cells, in studies using the Caco-2 cell line (45, 47).

Additionally, HIV infection dramatically depletes intestinal CD4^+^ T-cells, which plays a prominent role in the mucosal immunity of the gut barrier (35, 36, 48). The gut-associated lymphoid tissue (GALT) is deemed to be the largest lymphoid organ in the human body (49), within which about 70% of CD4^+^ T-cells express the C-C chemokine receptor type 5 (CCR5), a vital co-receptor for HIV entry into cells. In contrast, only 20% of peripheral blood CD4^+^ T-cells express CCR5 (16, 17, 50). Therefore, GALT can be seen to be the primary target and replication site for HIV, and CD4+ T-cells present in gut tissue are largely depleted after HIV infection. One subset of CD4^+^ T-cells, the T helper 17 (Th17) cell, promotes neutrophil recruitment and secretes antimicrobial peptides, interleukin 17 (IL-17), and interleukin 22 (IL-22) (36, 51). The depletion of Th17 cells may thus result in microbial overgrowth as well as the inhibition of epithelial regeneration (35, 51, 52). One study in rhesus macaques observed that depletion of mucosal Th17 cells by SIV infection promotes dissemination of gut *Salmonella typhimurium* into the peripheral blood (53).

Numerous studies have now shown that HIV infection is associated with intestinal microbiota dysbiosis, which has been considered to be the underlying factor in a diverse range of pathological processes (54–59). Specifically, in PLWH potential pathogens such as *Enterobacteriaceae* and *Erysipelotrichaceae* are enriched, while “protective” intestinal bacteria such as *Bacteroidaceae*, *Ruminococcaceae*, and *Akkermansia muciniphila* (*A. muciniphila*) are depleted (54, 60). *A. muciniphila* is a symbiont in the intestine and has been observed to thicken the mucus layer and fortify the integrity of epithelium (60–62). *Bifidobacteria* and *Lactobacillus* are considered to be “beneficial” microorganisms and, are reduced in PLWH (63), and past clinical trials have shown that supplementation with *Bifidobacteria*- and/or *Lactobacillus*-rich drugs may reduce levels of C-reactive protein (CRP), IL-6, and CD4^+^ T-cell activation in HIV infection (64–67). Butyrate is a major short-chain fatty acid (SCFA) in the gut, which serves as an energy source for epithelial cells, and promotes epithelial barrier integrity (68, 69). HIV infection reduces the frequency of butyrate-producing bacterial genera (e.g., *Roseburia*, *Coprococcus*, *Faecalibacterium prausnitzii*, and *Eubacterium rectale*) (53, 70, 71). Moreover, multiple past studies have shown the Proteobacteria phylum to be more abundant in HIV-infected individuals (55, 72–75), and includes several pathogens, such as *Pseudomonas* (56), *Desulfovibrio* (75), and *Shigella* (76). *Pseudomonas* is known to be capable of impairing host mucus production (77, 78), and *Shigella* has been shown to induce disruption of tight junctions (76, 79). Additionally, fungal communities are also significantly altered in PLWH (80). The abundance of *Candida albicans* (*C. albicans*), an opportunistic pathogen, is increased in the intestines of PLWH (80). Invasion by *C. albicans* actively contributes to enterocyte damage, with consequent cell death (81–83). *Clostridium difficile* (*C. difficile*) -related diarrhea is also common in PLWH (84, 85), and toxins from *C. difficile* (TcdA and TcdB) may disrupt intestinal tight junction integrity, thus promoting intestinal permeability (86–88).

Once the gut barrier is impaired by the collective and cumulative contributions of the preceding factors, products originating from both gut epithelial cells and the various gut microorganisms translocate into the blood. It is thus practicable and convenient to directly measure these circulating products (**Table 1**) in blood as the surrogates of gut damage and microbial translocation.

**Table 1:** Biomarkers for gut damage and microbial translocation in PLWH.

Biomarker	Origin	Design	Patient information	Change in PLWH	Mean value	Country	Reference
I-FABP	Host→ epithelia	A single center prospective study	93 ART-treated participants vs. 52 uninfected controllers	I-FABP is elevated in PLWH compared to HIV-negative individuals.	1581 vs. 1010 pg/mL	Canada	(89)
		A single center cross-sectional study	149 people with chronic HIV vs. 10 elite controllers	I-FABP is elevated in chronic HIV-infected patients compared to elite patients	3458 vs. 1947 pg/mL	USA	(90)
		A single center cross-sectional study	19 immune non-responders vs. 20 immune responders	Immune non-responders had higher levels of I-FABP than immune responders.	2089 vs. 1279 pg/mL	Norway	(91)
Zonulin	Host→ epithelia	A single center cross-sectional study	57 children with perinatally HIV infection vs. 56 HIV− children	Zonulin is elevated in PLWH compared to HIV-negative individuals.	10.95 vs. 5.54 ng/mL	Uganda	(92)
		A single center cross-sectional study	40 primary HIV+ patients vs. chronic HIV infected individuals	Zonulin is elevated in primary HIV-infected patients with high viral replication, compared to chronic HIV infected patients.	11.74 vs. 6.41 ng/mL	Mozambique	(93)
REG3α	Host→ Paneth cell	A single center prospective study	93 ART-treated participants vs. 52 uninfected controls	REG3α is elevated in PLWH compared to HIV-negative individuals.	2680 vs. 2059 pg/mL	Canada	(28)
		A single center cross-sectional study	19 immune non-responders vs. 20 immune responders	Immune non-responders have higher levels of REG3α than immune responders.	7196 vs. 4811 pg/mL	Norway	(91)
Citrulline	Host→ epithelia	A single center cross-sectional study	44 PLWH and 106 HIV-negative individuals	Citrulline is decreased in PLWH compared to HIV-negative individuals.	19 vs. 27 μmol/L	UK	(94)
DAO	Host→ epithelia	A single center prospective cohort study	20 patients with anticancer drug treatment	DAO was not reported in PLWH, DAO is elevated in IBD and anticancer drug treatment.	unavailable	Japan	(95)
LPS	Microorganism→ bacteria	A single center cross-sectional study	33 HIV-infected individuals vs. 31 HIV-negative individuals	LPS levels are mildly increased in acute/early HIV-infected individuals compared to HIV-negative individuals.	71 vs. 28 pg/mL	USA	(2)
		A single center cross-sectional study	24 immune non-responders vs. 11 immune responders	Compared to immunological responders, immunological non-responders had higher LPS levels.	45 vs. 29 pg/ml	Italy	(96)
		A single center cross-sectional study	14 elite controllers vs. 31 HIV-negative individuals	LPS levels are increased in elite controllers compared to HIV-negative individuals.	61 vs. 28 pg/mL	USA	(97)
16S rDNA	Microorganism→ bacteria	A single center cross-sectional study	19 HIV-infected individuals vs. 15 HIV-negative individuals.	16S rDNA levels are increased in HIV-infected individuals compared to HIV-negative individuals.	132.5 vs. 5 copies/μL	USA	(98)
		A single center randomized equivalence study	48 IRIS individuals vs. 93 non-IRIS individuals	16S rDNA levels are higher in participants with immune reconstitution inflammatory syndrome (IRIS) than that in non-IRIS patients.	32 vs. 58 copies/μL	USA	(99)
BDG	Microorganism→ fungi	A single center cross-sectional study	53 early HIV+ individuals vs. 42 HIV-negative individuals	BDG levels are increased in early HIV-infected individuals compared to HIV-negative individuals.	67.9 vs. 20.4 pg/mL	Canada	(44)
		A single center cross-sectional study	93 chronic HIV+ individuals vs. 53 early HIV+ individuals	BDG levels in chronic HIV-infected individuals are higher than in early HIV-infected patients.	91.86 vs. 68.72 pg/mL	Canada	(44)
		A single center cross-sectional study	57 children with perinatally HIV infection vs. 59 HIV−exposed but uninfected children	Compared with HIV-exposed but uninfected children, children with perinatally acquired HIV had higher BDG levels.	199.5 vs. 128,8 pg/mL	Uganda	(92)
Flagellin	Microorganism→ bacteria	A single center cross-sectional study	51 PLWH vs. 19 HIV-negative individuals	Anti-flagellin IgG levels are elevated in PLWH compared to HIV-negative individuals.	unavailable	Sweden	(100)
D-lactate	Microorganism→ bacteria	A single center cross-sectional study	29 active stage CD vs. 30 remission stage CD	D-lactate was not reported in PLWH, D-lactate is elevated in CD and gastrointestinal failure.	16.08 vs. 11.16 mg/L	China	(101)

→ symbol means “which specifically is”. The biomarkers originate from the host or microorganisms, which specifically are epithelia, bacteria or fungi.

## Plasma markers of gut damage and microbial translocation

### Biomarkers originating from host gut tissue

#### I-FABP

I-FABP is a member of the FABP family, has a molecular weight of approximately 15 kilodaltons (kDa), and plays a key role in the transportation and metabolism of long-chain fatty acids. It is specific to and abundant in the epithelial cells of the small intestine (102). Upon intestinal mucosal injury, I-FABP is released from epithelial cells into the circulating blood, and levels of plasma I-FABP have been reported to correlate with the severity of intestinal disease [e.g., ulcerative colitis, Crohn’s disease (CD), mesenteric ischemia, coeliac disease], and is considered a marker of intestinal epithelial cell tight junction disruption and cell death (103–107).

I-FABP has been shown to be an indicator of intestinal damage in PLWH. One prospective trial of ART-naïve HIV-infected individuals observed a remarkable elevation in I-FABP from baseline to week 96, and this elevation correlates with viral replication (108). Sustained effective ART significantly reduces immune activation and tends to ameliorate peripheral T-cell immunophenotypic imbalances; however, this positive and beneficial immunological response to targeted drug therapy is observed to be insufficient to adequately and comprehensively restore intestinal permeability and integrity, and plasma I-FABP levels remain markedly elevated subsequent to ART treatment (109). In chronic HIV-infected patients, I-FABP is increased to a much greater degree than in elite controllers and correlates with nutritional intake and body composition (body mass index, visceral and subcutaneous adipose tissue distribution) in HIV progressors (90). As an intestinal epithelial barrier marker, I-FABP may also act as a predictor of mortality in treated HIV-infected patients. Hunt et al., reported that, similar to other markers of intestinal barrier integrity (Zonulin-1) and some inflammatory factors, I-FABP strongly predicts mortality in treated HIV-infected individuals, with higher plasma I-FABP levels being associated with higher soluble CD14 (sCD14) levels, kynurenine-to-tryptophan (K/T) ratios, IL-6, and D-dimer levels, and lower proximal CD4^+^ T-cell counts (110). It is also worth noting that levels of plasma I-FABP have also been reported to correlate with other non-inflammatory factors (111–113). Our previous study observed that levels of I-FABP were affected by circadian rhythm, and plasma levels of I-FABP at 16h00 were significantly lower, compared to levels at 08h00 and 04h00 (111). Auinger et al., have demonstrated that I-FABP expression significantly correlates with fatty acid intake in food as well as I-FABP gene polymorphisms (112).

#### Zonulin

Zonulin is a protein with a molecular mass of 47 kDa, is specifically expressed at the cell surface and secreted by the intestinal epithelium, and is the only physiological enzyme discovered thus far that is known to regulate intestinal permeability *via* reversibly disassembling the intercellular tight junctions between intestinal epithelial cells (114–116). Serum zonulin has been reported to be elevated in inflammatory and autoimmune disorders [e.g., type 1 diabetes, inflammatory bowel disease, ankylosing spondylitis (AS), and CD], and its levels correlate with the opening of tight junctions and increased intestinal permeability when zonulin production is dysregulated (117–119). It has also been observed that the synthetic TJ regulator larazotide (which acts in an inhibitory capacity) blocks zonulin enzyme activity with excellent efficacy in type 1 diabetes and celiac disease (118, 120, 121).

The zonulin-driven intestinal cellular immune response has been validated during *in vitro* experiments (122). When the small intestine is exposed to microbes, there is an increase in the secretion of zonulin from the intestinal lumen. Zonulin disassembles TJs by separating the zonula occludens-1 protein from the TJ complexes, decreasing transepithelial electrical resistance (TEER), and increasing permeability (122). Likewise, plasma zonulin levels are elevated in PLWH and are associated with monocyte and T-cell activation (92, 123). Perinatally HIV-infected infants show increased circulating levels of zonulin despite viral suppression by treatment with early ART, and these levels are significantly higher than in HIV-exposed but uninfected infants at 5 months of age (124). Compared to chronically HIV-infected patients, plasma zonulin levels in primary HIV infection (PHI) are significantly elevated and correlate with high viral replication. Plasma zonulin also demonstrates the best accuracy to identify PHI among HIV-infected individuals {AUC=0.85 [95% CI 0.75-0.94]}. Using a cutoff value of plasma zonulin >8.75 ng/mL, the model identified PHI with 87.7% sensitivity and 69.2% specificity (93).

Additionally, elevated zonulin levels have also been associated with disease progression in PLWH. Hunt et al., reported that zonulin has the strong capacity to predict mortality in treated PLWH who had an AIDS history (110). In perinatally HIV-infected children with a history of breastfeeding, zonulin is associated with multiple markers of systemic inflammation, including CRP, IL-6, and D-dimer (92). Improvement and amelioration of the intestinal barrier *via* serum bovine immunoglobulin significantly reduces circulating levels of I-FABP and zonulin, and systemic inflammation is also ameliorated (125).

#### REG3α

REG3α, is a C-type lectin antimicrobial peptide secreted by Paneth cells in the gut lumen, and helps contain bacterial infection by binding to peptidoglycans in the cell wall of certain bacteria, and has the capacity to kill some gram-positive bacteria (126, 127). REG3α also helps maintain intestinal barrier integrity by reducing apoptosis of intestinal epithelial cells (128). When the integrity of the intestinal epithelial barrier is disrupted, REG3α crosses through the epithelium, translocates to the lamina propria, and subsequently enters into the systemic circulation (28, 129). Thus, circulating REG3α levels are considered to be a marker of intestinal permeability.

REG3α has long been proposed as a biomarker of intestinal epithelial damage in multiple inflammatory diseases in which plasma REG3α levels are significantly elevated, such as CD, celiac disease, ulcerative colitis, nonalcoholic steatohepatitis, and gastrointestinal graft-versus-host disease (129–133). Heavy alcohol consumption is known to disrupt the integrity of the gut epithelium. Yang et al., used REG3α and Trefoil factor 3 as biomarkers to assess intestinal damage and microbial translocation in patients with alcoholic hepatitis, and observed that circulating levels of these markers are highly elevated, and differentially correlates with disease severity, sCD14 levels, and IL-6 levels (134). Darnaud et al., studied the mechanism by which REG3α maintains intestinal homeostasis and affects inflammatory responses in genetically engineered C57BL/6 mice. The preceding authors observed that REG3α is a potent reactive oxygen species (ROS) scavenger that reduces oxidative stress and inflammatory responses in intestinal epithelial cells, reduces host susceptibility to colitis, and alters the murine gut microflora composition (inducing an increase in *Clostridium* and a decrease in *Bacteroides* and *Proteus*) by reducing ROS levels (135).

Given that REG3α is a reliable marker of intestinal barrier damage, REG3α has been also used in HIV patients to assess the degree of gut damage and systemic immune activation. As reported by Isnard et al., plasma REG3α levels are elevated in untreated and ART-treated PLWH (including elite controllers), when compared to HIV-negative individuals. In contrast, plasma REG3α levels are decreased in PLWH who initiate ART, compared with untreated patients. REG3α levels also negatively correlate with CD4^+^ T-cell counts and CD4^+^/CD8^+^ ratios, and positively correlate with HIV viral load, fungal translocation products, and inflammatory markers in all PLWH (28). Immune non-responders (INRs) are PLWH who fail to adequately restore CD4^+^ T-cell numbers after effective ART and are known to have irreversibly impaired intestinal mucosal barrier function. It has been observed that INRs have higher levels of plasma I-FABP and REG3α, compared to immune responders (136), and that mucosal CD4^+^ T-cells positively correlate with I-FABP and REG3α (91).

#### Citrulline

Citrulline is a non-essential amino acid synthesized from glutamine, and plays a role associated with inflammatory disease (137–142). In several organ exclusion experiments, it has been observed that most citrulline is synthesized in the intestinal epithelium, and subsequently enters into the blood circulation; thus, the intestinal epithelium is the main source of circulating citrulline under physiological conditions (138, 143–145). When intestinal epithelial injury occurs, the production of citrulline decreases, and consequently, in contrast to the trend seen for other biomarkers, the plasma concentration of citrulline decreases.

Circulating citrulline has been used to estimate the degree of intestinal injury, and circulating levels negatively correlate with disease severity in intestinal enteropathies (94, 137, 146, 147). Crenn et al., analyzed the relationship between plasma citrulline concentrations and villous atrophy in celiac disease (148). Their results showed that plasma citrulline concentration is lower in patients with villous atrophy than in healthy subjects, and negatively correlates with the severity of villous atrophy. The results of an investigation in pediatric and adolescent cases of CD by Diamanti et al., showed that CD patients have a reduced concentration of plasma citrulline compared to controls, and that plasma citrulline levels are significantly lower in patients with small bowel localization of CD than in patients with ileo-colon disease (149). Nuzzo et al., analyzed plasma citrulline concentrations in acute mesenteric ischemia (AMI), and plasma citrulline concentrations were observed to be significantly lower in AMI patients compared to controls; however, its practicality for the diagnosis of AMI was found to not be satisfactory, with an area under the receiver operating curve (AUROC) sensitivity and specificity of 0.68, 56%, and 84%, respectively (137). Kulu et al., also observed similar results, with plasma citrulline concentrations in AMI patients being lower, and the AUROC sensitivity and specificity for the diagnosis of AMI were 0.72, 39%, and 100%, respectively, with the best cut-off value being 15.82 µmol/L (150). Furthermore, Fragkos et al., used a meta-analytical method to assess citrulline as a biomarker of gut damage (147), and their results observed that plasma citrulline negatively correlates with disease severity in intestinal enteropathies. The preceding authors have advocated for the use of citrulline as a marker for acute and chronic intestinal insufficiency. In PLWH, Papadia et al., reported that median citrulline levels in HIV positive individuals is significantly lower than that in HIV-negative individuals, and that there are statistically significant correlations between citrulline and villous atrophy in HIV positive individuals (94). Thus, citrulline has been identified as a biomarker for intestinal injury; however, further studies are warranted in order to validate its accuracy among different subgroups of PLWH.

#### Diamine oxidase

Human diamine oxidase (hDAO) is encoded by the amine oxidase copper containing 1 (AOC1) gene, located on chromosome 7q35 (151). DAO is mainly expressed in the human intestinal mucosal epithelium, the placenta, and the kidney, and catalyzes the oxidation of diamines such as histamine, putrescine, and cadaverine. High activity of this enzyme is found in the mature upper villus cells of the intestinal mucosa, and the intestine is the sole source of plasma diamine oxidase (152–156). DAO is normally present in exceedingly small quantities in the systemic circulation, is stable in the circulation, and high plasma levels are associated with poor integrity of the intestinal mucosa (156, 157).

DAO has been used as a serum marker of intestinal injury (156, 157). It has been reported that plasma DAO activity is associated with the histological and biochemical changes indicative of injury and recovery in a rat model of intestinal injury (155). DAO activity has also been observed in some diseases causing intestinal impairment in humans, including irritable bowel syndrome (IBS) and inflammatory bowel disease (IBD) (158–161). Meng et al., studied the intestinal injury induced by high dose methotrexate in children with acute lymphoblastic leukemia (158), and they observed that levels of plasma LPS and DAO at 1h, 24h, and 44h gradually increased after treatment (158). Zhang et al., reported a similar tendency in DAO levels in patients with heatstroke (160). Additionally, Ji and colleagues observed higher DAO activity levels in 60 patients with IBS compared to 20 healthy controls, and DAO activity levels correlated with disease severity (162). It is worth noting, however, that some investigations observed decreased plasma DAO levels in some severe instances of intestinal disease, such as IBD (161, 163), and anticancer drug treatment (95, 164). A probable reason that may explain these contrasting observations could be related to the fact that DAO-producing intestinal epithelial cells were significantly reduced in the latter specific instances (163, 165, 166). Moreover, several factors may affect DAO activity, such as genetic variability, the pH-value and temperature of the surrounding milieu, and medication use (167–169). Ayuso et al., identified three variants (Thr16Met, Ser332Phe, and His645Asp) of the AOC1 gene, and reported that individuals carrying the His645Asp variant displayed lower serum DAO activity as compared with noncarriers, with a significant gene-dose effect (168). *In vitro* experimental results have observed a potent DAO inhibitory effect (of greater than 90%) associated with chloroquine and clavulanic acid (167).

It is, therefore, suggested that DAO may be used as a candidate biomarker to evaluate the extent of intestinal injury when few other factors may be discernable, especially in the initial stages of intestinal injury.

### Biomarkers originating from gut microorganisms

#### LPS, sCD14, and 16S rDNA

Lipopolysaccharide (LPS), a critical component of the cell wall of gram-negative bacteria, consists of hydrophobic lipids and hydrophilic sugars, and is soluble in both water and lipids, thus facilitating diffusion (170, 171). The intestinal barrier prevents microorganisms and their products, such as LPS, from entering into the systemic circulation (36, 48). In PLWH, however, the disrupted intestinal barrier together with an increased permeability allows LPS to translocate into the blood circulation (36). In addition, when the monocyte/macrophage receptor for LPS, i.e., CD14, binds with circulating LPS, this induces activation and systemic inflammation (172, 173). CD14 is cleaved and released upon cell activation. Plasma LPS and sCD14 levels are increased in PLWH (36), and plasma levels of sCD14 have been generally shown to positively correlate with circulating LPS levels (174). Thus, circulating LPS and sCD14 have been frequently utilized as biomarkers of bacterial translocation, and have been observed to be associated with systemic immune activation and HIV disease progression (34, 171, 173, 175, 176). Nevertheless, some studies have also reported that LPS either negatively correlates with or does not correlate with sCD14 at all (177–179). This ambiguity may relate either to the CD14 genomic polymorphism (180), multifarious LPSs from different bacteria (181), a different extent of influence from ART, or opportunistic coinfections in these patients (177).

Immunohistochemistry results in SIV-infected rhesus macaques illustrate multifocal compromises and epithelial breaches in the normally intact epithelial barrier in gut tissues. In concert with this, quantitative image analysis of microbial translocation has revealed the emergence of LPS in the gut as well as parenteral tissue of rhesus macaques (1). Numerous studies have shown that levels of LPS and sCD14 are raised in PLWH (2, 34, 96, 175). Brenchley et al., reported that LPS levels are mildly increased in acute/early HIV-infected individuals, compared to HIV-uninfected individuals (2). However, significantly higher LPS levels are observed in chronic HIV-infected individuals, and sCD14 levels are raised in all cohorts of PLWH (2). LPS levels have also been associated with increased activation of activated CD4^+^ and CD8^+^ T-cells and plasma Interferon-alpha (IFN-α) levels (2). Even elite controllers have higher LPS levels than HIV-negative individuals, and the higher plasma LPS levels are associated with higher activated CD8^+^ T-cell counts (97). Furthermore, research by Marchetti et al., observed that compared with immunological responders, immunological non-responders, additionally, had higher LPS levels, which correlates significantly with frequencies of activated CD4^+^ and CD8^+^ T-cells (96).

A robust association has also been observed between clinical outcomes and levels of sCD14 and LPS (182–184). A study by Marchetti et al., observed that in a cohort of 379 HIV-infected individuals, circulating LPS level is a strong predictor of disease progression, independently of CD4^+^ T-cell counts and plasma viral load counts (175). PLWH with higher LPS levels showed a substantially accelerated disease progression rate, with a median time to clinical event of 1.5 years, compared with 4 years for patients with lower LPS levels (175). Jumare and colleagues observed that compared with uninfected controls, plasma levels of sCD14 were significantly higher in PLWH, and among PLWH, those with neurocognitive impairment had significantly higher sCD14 levels compared with neurocognitively unimpaired individuals (182).

Presence of circulating fragments of microbial DNA is generally accepted to be valid evidence for bacterial translocation (185, 186). DNA sequences encoding bacterial ribosomal 16S RNA (16S rDNA) corresponds to rRNA on bacterial chromosomes, with a length of about 1542 base pairs (bp) (187). 16S rDNA exists in all bacterial chromosomal genes, with homologous functions, and the oldest of these genes (known as “bacterial fossils”) are highly conserved in structure and function (188). As an important bacterial product, 16S rDNA of gut origin is highly likely to translocate into the systemic circulation in the presence of compromised gut integrity (189). Thus, measurement of 16S rDNA levels in plasma has the capacity to effectively reflect levels of microbial translocation (186, 189, 190).

By quantitative PCR (qPCR) determination of 16S rDNA fragments, Jiang et al., observed that plasma 16S rDNA levels in HIV-infected individuals are significantly higher than in uninfected individuals, and correlates with LPS levels (21, 178, 190). Plasma 16S rDNA level increases with duration of HIV infection (98), and treatment with ART may reduce, but does not fully eliminate plasma levels of bacterial 16S rDNA (109). Higher levels of 16S rDNA during treatment are strongly associated with higher T-cell activation and lower CD4^+^ T-cell recovery, regardless of plasma HIV RNA viral load (21). In the context of non-human primate lentiviral infection, 16S rDNA levels and CD8^+^ T-cell activation inversely correlate with the Th17/regulatory T-cell (Th17/Treg) ratio, suggesting that the extent of microbial translocation and T-cell activation in progressive HIV disease is closely related to skewed maturation along the TH17/Treg axis (191). These findings highlight the importance of microbial translocation markers such as LPS and sCD14 in the context of immunodeficiency and T-cell homeostasis in chronic HIV infection (2, 21, 192).

Plasma 16S rDNA is strongly associated with persistent immune activation in HIV disease. Among HIV-Hepatitis C virus (HCV) co-infected patients, bacterial 16S rDNA levels are notably higher than in the ART-controlled HIV-positive group, and 16S rDNA levels have been seen to increase with duration of HIV infection (98, 193). In HIV-positive patients with neuro-inflammation, Jaime et al., observed a significant correlation between plasma concentrations of 16S rDNA and increased expression of translocated proteins in brain regions with markedly active microglia, such as the basal ganglia and the globus pallidus (194). Plasma 16S rDNA concentrations are also associated with increased white matter tract density (194). One study by Bossola et al. (195), observed that plasma levels of bacteria-derived 16S rDNA in whole blood are statistically significantly associated with higher levels of CRP and IL-6 in patients undergoing chronic hemodialysis. Furthermore, 16S rDNA may bind to Toll-like receptors (TLR) and stimulate immune cells (196). These induce Natural Killer (NK) cell activity and the release of IFN-γ, tumor necrosis factor alpha (TNF-α), and IL-6 from mononuclear cells (197–200). Similarly, in treated HIV-infected patients, higher levels of inflammatory markers (IL-6 and TNF-α) are associated with microbial translocation (16S rDNA, sCD14) and previous cardiovascular events (201).

#### BDG

Fungi are also an integral component of the gut microbiome and is second only to bacterial frequency in terms of numbers of organisms (80, 202, 203). Similar to translocation of bacterial products, higher circulating levels of fungal products have also been found in PLWH (44, 123, 204). As a major component of most fungal cell walls, (1, 3) β-D-Glucan (BDG) has now been validated as a fungal translocation biomarker (27, 29), and is contemporarily used for the clinical diagnosis of invasive fungal infections (IFI) (205, 206).

Leelahavanichkul et al., tested serum BDG levels in several murine models of gastrointestinal leakage (including dextran sulfate solution administration, LPS injection, and cecal ligation and puncture sepsis), and observed increased levels of the fungal product, BDG, in the systemic circulation (207). Morris et al., first reported that the fungal product, BDG, is present in the blood of PLWH, and that those individuals with higher plasma BDG are associated with lower CD4^+^ T-cell counts, a higher viral load, and cardiopulmonary comorbidity (208). Subsequently, other investigators also reported higher BDG levels in PLWH (92, 209–211). An investigation by Mehraj et al., showed that, in a similar manner to LPS levels indicating translocation of bacterial products, plasma BDG levels are elevated in all HIV-positive patients without IFI, compared with HIV-negative controls (44). The preceding group prospectively assessed the levels of BDG in early and chronic HIV infection and observed increasing levels over time, in absence of treatment (44). Similarly, a cross-sectional study conducted in Ugandan children showed that compared with HIV-exposed uninfected children, those who acquired HIV perinatally had higher plasma BDG levels that correlated with IL-6 and D-dimer levels (92). A longitudinal investigation in 451 participants who were ART-naïve at baseline showed that BDG levels had decreased after 48 weeks of ART treatment, and higher BDG levels are associated with increased risk of non-AIDS events (209). Hoenigl et al., reported that plasma BDG concentrations are not affected by plant BDG-rich food, further indicating that translocated BDG may be deemed as a reliable marker of intestinal fungal translocation (212). These studies indicate BDG may function as a potential biomarker to indicate whether to initiate antifungal prophylaxis in the early stages of HIV infection, or not.

Higher blood BDG levels have also been reported to correlate with immune activation and risk of developing non-AIDS comorbidities (27, 29, 44, 213). Among PLWH on ART, higher plasma BDG levels are associated with higher levels of activated CD4^+^ and CD8^+^ T-cells, and positively correlates with the K/T ratio (44), which is linked to gut damage and bacterial translocation during HIV infection (214–216). Weiner et al., reported that higher BDG levels are associated with the inflammatory cytokines sCD14, IP-10, D-dimer, and sCD163 (213). A study by Hoenigl et al., reported that higher plasma BDG levels were related significantly to worse neurocognitive performance among HIV-infected individuals with suppressed viral loads (217). An investigation by Isnard et al., observed the association between elevated plasma BDG levels and subclinical coronary atherosclerotic plaques in PLWH. Interestingly, BDG levels (and not LPS levels) were elevated significantly in ART-treated PLWH with subclinical coronary atherosclerosis and correlates with total plaque volume (89).

#### Bacterial flagellin

Flagellin is a structural protein present on the flagella of most motile bacteria in the intestine. Flagellin is recognized and bound by TLR5 on the cell membranes of immune cells, resulting in activation of these cells (218). Bacterial flagellin acts as a pathogen-associated molecular pattern (PAMP) with strong antigenicity, and plays a significant role under conditions of gut damage, including in inflammatory bowel disease (218–220), necrotizing enterocolitis (221), and diarrheal diseases (222, 223). Flagellin is considered to be the major immune antigen in Crohn’s disease, with bacterial flagellin antibody detected in approximately half of Crohn’s disease patients (224, 225). Anti-flagellin antibodies recognize the TLR5 and PATJ (PALS-1-associated tight junction protein) and induce monocyte activation and increased intestinal permeability in Crohn’s disease (226). Svärd et al., reported that circulating flagellin correlates with anti-flagellin levels, and is associated with activation of monocytes in chronic HIV-1-infected individuals (227).

Flagellin promotes virus entry into epithelial cells (228), and enhances HIV-1 induced mucosal immunity in the intestine (229). *In vitro*, the flagellin/TLR5 complex has been observed to directly trigger viral replication in HIV-infected cells (100). Thibault and colleagues have reported that flagellin, by itself, is able to activate latent HIV-1 provirus in T-lymphoid cells and provoke virus gene expression in central memory CD4^+^ T-cells (230). A study by Brichacek et al., showed that flagellin enhances HIV-1 replication, and activation of CD4^+^ T-cells in tonsillar tissue *ex vivo* (231). In PLWH, elevated levels of anti-flagellin immunoglobulin G (IgG) are present in ART-naïve HIV infected individuals, as compared to HIV-negative controls, and after ART treatment, the level of anti-flagellin IgG decreases (100, 232, 233). The outcomes of these studies imply that in PLWH, flagellin may accelerate the depletion of immune cells in the intestinal mucosa, which in turn worsens the integrity of the intestinal barrier and further encourages the entry of flagellin into the circulation.

#### D-lactate

D-lactate (D-LA) is a product released by bacteria residing in the human gut, and is not known to be produced in other tissues; thus, the D-LA present in plasma can be assumed to originate from the gastrointestinal tract (234–237). In other words, plasma D-LA levels may also be used as a surrogate for the degree of gut permeability and epithelial damage.

Increased plasma D-LA and LPS levels have been observed in some disease models that lead to intestinal mucosal damage. Increased D-LA levels are associated with diseases which induce severe intestinal injury (238–240). Cai et al., observed and reported on the correlation between D-LA and Crohn’s disease activity. Their study found that serum D-LA levels in patients with active Crohn’s disease and that of those in disease remission were 16.08 ± 4.8 mg/L and 11.16 ± 3.17 mg/L, respectively. Serum D-lactate levels were significantly higher in the active phase compared to the remission phase of Crohn’s disease (241). Teng et al., observed that serum D-LA is associated with acute gastrointestinal injury (AGI) and failure in critically ill patients. D-LA and LPS levels were observed to be higher in patients in the gastrointestinal dysfunction (GID) group or the gastrointestinal failure (GIF) group, than in the healthy control group (101).

Similarly, plasma D-LA can also be used as a marker to predict the degree of damage to the intestinal barrier in HIV patients, and D-LA is associated with recovery of CD4^+^ T-cell counts. HIV-positive individuals who are immunological non-responders despite ART are prone to malnutrition and compromised gut barriers, which further exacerbates chronic immune activation and inflammation (242). Geng et al., conducted enteral nutritional intervention in these populations and found that serum D-LA and LPS levels were significantly lower in PLWH, with good immune reconstitution after intervention, compared with levels of these markers pre-intervention. Also, D-LA levels negatively correlate with the recovery of CD4^+^ T-cells, and positively correlate with levels of the inflammatory factor, IL-1β (243).

## Plasma biomarkers are widely used to assess the adequacy of therapeutic strategies in PLWH

In view of the significant structural and functional changes caused by HIV infection to the intestinal epithelial barrier, exploration of methods to potentially restore intestinal barrier function is emerging as a research priority, in addition to improvements to ART for PLWH (**Table 2**).

**Table 2:** Potential therapeutic strategies to improve the integrity of the intestinal barrier.

Therapeutic strategy	Model	Design	Intervention	Change in PLWH	Reference
Target gut microbial composition					
Probiotics	PLWH with ART (20 cases)	A longitudinal pilot study	Arm I: supplementation with probiotic	The activation of CD4+ T-cells and levels of sCD14, LBP, and CRP were decreased after probiotic supplementation.	(64)
Prebiotics	ART-naïve PLWH (57 cases)	A longitudinal pilot study	Arm I: supplementation with prebiotic	Prebiotic improves the gut microbiota composition, reduces sCD14 level and CD4+ T-cell activation, and improves NK cell activity.	(66)
Antibiotics	PLWH (26 cases)	A longitudinal pilot study	Arm I: treatment with trimethoprim and sulfamethoxazole (TMP-SMX)	Concomitant use of ART and TMP-SMX reduces microbial translocation markers LBP and sCD14	(244)
FMT	PLWH with ART (30 cases)	A longitudinal randomized study	Arm I: fecal microbiota capsules Arm II: placebo	The alpha diversity of the constituent microbiota of the gut microbiome increases after FMT.	(245)
Target microbial products and intestinal epithelial					
Sevelamer	ART-naïve PLWH (36 cases)	A longitudinal pilot study	Arm I: sevelamer carbonate orally 3 times daily for 8 weeks	Sevelamer does not significantly change markers of microbial translocation, inflammation, or T-cell activation.	(246)
Larazotide acetate	Patients with CD (20 cases)	A double-blind, randomized placebo-controlled study	Arm I: larazotide acetate Arm II: placebo	Larazotide acetate significantly reduces intestinal permeability.	(121)
Target immune activation					
Mesalazine	UC and/or HIV infected patients (26 cases)	A cross-sectional study	Arm I: HIV+/UC+ Arm II: HIV+/UC- Arm III: HIV-/UC+ All patients with UC were treated with oral mesalazine.	Plasma levels of sCD14 and I-FABP in HIV-infected patients with mesalazine-treated UC reduced	(247)
Glucocorticoids	PLWH (101 cases)	An observational study	Arm I: untreated HIV patients Arm II: HIV patients treated with prednisolone Arm III: HIV patients treated with ART Arm IV: HIV patients treated with Prednisolone/ART Arm V: no treatment, elite controllers	Low-dose prednisolone significantly decreases levels of sCD14, LBP, and suPAR antigen compared to untreated patients	(248)

Therapeutic strategies that target gut microbial composition to potentially restore the physiological gut microbiome and gut barrier integrity have been evaluated in the past. At present, a number of methods capable of altering and modifying gut microbial composition have been reported, including supplementation with probiotics, prebiotics, and synbiotics, fecal microbiota transplantation (FMT), and antibiotic use; however, it remains a confounding exercise to coherently interpret the effects and consequences of these methods on gut microbial composition because of the heterogeneity of recent studies with respect to study design, participant ethnicity, HIV status, ART regimen used, etc. Probiotics and prebiotics are deemed as effective adjuvant therapeutic strategies for PLWH. D’Ettorre et al., conducted a clinical trial of oral probiotics (with an abundance of *Streptococcus salivarius* and *Bifidobacteria*) in ART-treated PLWH over 48 weeks, and microbial translocation and immune activation were evaluated by blood sCD14, LBP, and CRP levels. Results indicated that the activation of CD4^+^ T-cells and levels of sCD14, LBP, and CRP were all decreased after probiotic supplementation (64). A study with ART-naïve HIV-infected individuals showed that prebiotic supplementation (with an oligosaccharide mixture) improved bifidobacterial levels, and decreased *C. lituseburense*/*C. histolyticum* levels and sCD14 concentrations in plasma (66). Moreover, it has been reported that HIV infection induces a depletion of *A. muciniphila* in the intestine (60, 249). Supplementation with *A. muciniphila* can relieve the inflammation of chronic colitis (250, 251). Liu et al., assessed intestinal permeability in *A. muciniphila* treated mice with bone fractures by measuring plasma LPS and Fluorescein Isothiocyanate (FITC)-dextran, and reported that *A. muciniphila* treatment decreases LPS and FITC-dextran levels, and increases mRNA expression of tight junction proteins, including occludin, jam3, claudin-2, -3, and -15 (252). Hence, elevating the level of *A. muciniphila* in the gut appears to be a potentially effective treatment strategy to foster the integrity of the gut barrier to some extent. In addition, a recent study by Wang et al., has shown that an extract of the Chinese traditional medicine, Painong-San, alleviates colitis by upregulating the expression of tight junction proteins [claudin-1, occludin, and zonula occludens-1 (ZO-1)], and increases the abundance of probiotic organisms, including *Lactobacillus*, *Bifidobacterium*, and *A. muciniphila* in a murine model (253).

FMT is known to be an effective therapy for recurrent *C. difficile* infection through transplantation of fecal microbiota from a healthy donor into the gastrointestinal tract of a recipient (254–257). A study by Konturek et al., has shown that the serum level of pro-inflammatory cytokines (TNF-α, IL-1β, IL-6, IL-8, and IL-12) decreases significantly post FMT, and the abundance of beneficial bacterial species such as *Lactobacillaceae*, *Ruminococcaceae*, *Desulfovibrionaceae*, *Sutterellaceae*, and *Porphyromonodacea* increase after FMT in patients with *C. difficile* infection (254). Cheng et al., used serum DAO activity and D-lactate to assess the gut barrier in a gut-injured piglet model, and observed that serum DAO activity and D-lactate levels reduced significantly in FMT-treated piglets. FMT has also been observed to increase the protein expression of ZO-1 and occludin in the colonic mucosa, and to increase the abundance of beneficial bacteria, such as *Lactobacillus* and *Succinivibrio*, and decrease the abundance of *Enterobacteriaceae* and *Proteobacteria* (258). In PLWH, the safety of FMT was investigated by Vujkovic-Cvijin et al., and they observed that no serious adverse effects occurred during 24 weeks of follow-up after one-time FMT, and during the 8 weeks post-FMT, recipients demonstrate partial engraftment of the donor microbiota, and no differences in sCD14 levels were observed (259). In addition, in a pilot FMT study in HIV-infected individuals, a significant 0.5-fold decrease of I-FABP was observed in the FMT group, while no statistically significant decrease in sCD14 and LBP levels was detected. They also observed that there was a significant increase in the alpha diversity of the constituent microbiota of the gut microbiome, and an increase in several members of the *Lachnospiraceae* family, including *Anaerostipes* spp., *Blautia* spp., *Dorea* spp., and *Fusicatenibacter* spp. after FMT (245).

The use of antibiotics is a direct method to alter microbial composition in the gut. Administration of Rifaximin (a nonabsorbable antibiotic) combined with sulfasalazine (an anti-inflammatory drug), has been shown to decrease LPS and sCD14 in SIV-infected pigtailed macaques (260). However, a study by Tenorio et al., observed that there were no significant changes in LPS and sCD14 levels in Rifaximin-treated PLWH (261). Moreover, cotrimoxazole (trimethoprim + sulfamethoxazole) is commonly used to prevent *Pneumocystis jirovecii* infection in HIV-infected patients, and an investigation by Vesterbacka et al., has observed that levels of LPS and sCD14 show no additional reduction in PLWH who initiated ART together with cotrimoxazole in over two years of use (244).

Improving intestinal epithelial function and reducing the entry of microbial products into the bloodstream may also be useful. Sevelamer, a phosphate-lowering drug, has been reported to decrease circulating LPS levels in subjects with chronic kidney disease (262, 263), and also decreases LPS levels by 80% and reduces CRP levels by 78% in subjects on hemodialysis (264). In a SIV-infected pigtailed macaque model, Kristoff et al., assessed intestinal damage *via* plasma LPS and sCD14 levels, and revealed that sevelamer treatment (2400mg, 3 times per day) reduces LPS, sCD14, and SIV viral loads, and decreases the frequency of HLA^-^DR^+^CD38^+^CD8^+^ T-cells (265). However, Sandler et al., designed a clinical trial of sevelamer (1600mg, 3 times per day) administration over 8 weeks, and their results observed an absence of a statistically significant decrease in LPS and sCD14 levels in ART-naïve HIV-infected patients (246). Moreover, larazotide acetate (also called AT-1001), an inhibitor of zonulin, has been shown to inhibit TJ disassembly and dysfunction caused by endogenous and exogenous stimuli in intestinal epithelial cells (121, 266–268). *In vivo*, larazotide acetate significantly reduces the frequency of gastrointestinal symptoms, particularly diarrhea in coeliac disease subjects, and generates a 70% decrease of intestinal permeability (121). Glutamine is a major amino acid in the human body, and is a common substrate used by intestinal cells. Multiple lines of evidence indicate that glutamine regulates the expression of tight junction proteins (269, 270). The depletion of glutamine results in enterocyte atrophy and a subsequent increase in permeability of the intestinal barrier, and supplementation with glutamine has the potential capacity to promote enterocyte proliferation, fortify intestinal membrane integrity, and reduce microbial translocation (269, 271–273).

Immune activation induced by microbial translocation plays a vital role in gut damage. Microbial products may provoke pro-inflammatory responses by binding to numerous receptors i.e., the nucleotide-binding oligomerization domain, as well as multiple TLRs. These receptors are expressed by a number of immune cells, including monocytes, macrophages, and dendritic cells. Once the microbial substrates bind to the receptors, a signaling cascade is activated, subsequently inducing the secretion of many inflammatory cytokines, e.g., IL-1β, IL-6, TNF, and type I IFNs (36, 272, 274). Novel approaches targeting microbial products or their downstream effects, thus attenuating immune activation, may also be a potentially effective therapeutic strategy. The 5-ASA preparations (e.g. mesalazine, sulfasalazine) are clinically effective drugs for the treatment of IBD (which causes intestinal damage), and act by modulating several gut inflammatory pathways [including those associated with Peroxisome proliferator-activated receptor gamma (PPARγ), arachidonic acid and leukotriene biosynthesis, NF-kappaB (NF-κB), and mechanistic target of rapamycin (mTOR)] (275–279). In an SIV-infected animal model, Pandrea et al., used LPS and sCD14 to assess the levels of microbial translocation in SIV-infected pigtailed macaques, and their results indicate that LPS and sCD14 levels are significantly reduced in Rifaximin- and sulfasalazine-treated acutely SIV-infected pigtailed macaques; however, their use induces no statistically significant reductions in LPS and sCD14 levels in chronic SIV-infected pigtailed macaques (260). Furthermore, rifaximin and sulfasalazine treatment significantly reduces the levels of CD4^+^ and CD8^+^ T-cell activation and improves hypercoagulation in acute SIV-infected pigtailed macaques (260). In PLWH, a randomized crossover trial (280) of oral mesalazine for 12 weeks showed that, compared to placebo-treated subjects, plasma sCD14 levels did not significantly decrease, and there is no evidence of an effect of mesalazine on CD8^+^ and CD4^+^ T-cell activation, IL-6 levels, D-dimer levels, or the K/T ratio at any time point. However, an investigation by Michelini et al. (247), observed that, compared to HIV-infected patients, the plasma levels of sCD14 were significantly lower in mesalazine treated HIV-infected patients with ulcerative colitis; however, the levels of I-FABP were found to not be statistically different.

Furthermore, glucocorticoids (GCs, e.g. dexamethasone, betamethasone, prednisolone) are therapeutically used for their anti-inflammatory and immunosuppressive effects in clinical medicine (281). *In vitro* experiments indicate that GCs regulate the intestinal tight junction barrier in the intestinal epithelial Caco-2 cell line model (282, 283). Fische and colleagues (282) reported that dexamethasone induces a time- and dose-dependent increase in transepithelial electrical resistance on Caco-2 cell monolayers, which is an *in vitro* model of the intestinal epithelial barrier, and the expression of claudin 2 (which is involved in pore formation) was downregulated, while expression of claudin 4 (which contributes in the sealing of TJs) was elevated in the dexamethasone treated Caco-2 cell line. Similarly, prednisone treatment of patients with active Crohn’s disease demonstrates a significant reduction in intestinal permeability in the majority of treated individuals, as assessed by the lactulose-mannitol ratio (284). Nockher et al., reported that prednisolone suppressed expression and release of sCD14 *in vitro* and *in vivo* (285). An investigation by Kasang et al., in HIV^+^ patients observed that low-dose prednisolone significantly decreases levels of sCD14, LBP, and soluble urokinase plasminogen activated receptor (suPAR) in untreated patients; however, there were no significant changes in ART-treated patients (248). GCs have also been reported to decrease LPS-induced inflammatory responses (286–288), reduce HIV viral loads, and to postpone CD4^+^ T-cell loss (289, 290), and the progression to AIDS (291). However, these therapies remain experimental, and the utilization of GCs must be reserved for specific circumstances only and should be strictly controlled, as their use may raise multiple issues, including adverse effects (292, 293) and the interactions of GCs with antiretroviral-boosting agents (e.g., ritonavir and cobicistat) that are currently included in ART regimens (294, 295). The inflammatory cytokine TNF-α has been reported to increase epithelial cell shedding and increases intestinal epithelial tight junction permeability, along with enhancing gut permeability (283, 296, 297). The TNF-α inhibitors infliximab and adalimumab have been used in the treatment of IBD, and exert their effects by inhibiting the TNF pathway, thus decreasing inflammation and restoring mucosal integrity (298–301). A trial conducted in 23 patients with active CD showed that gut permeability, CRP, and the Crohn’s Disease Activity Index (CDAI) significantly decreased after a single infusion of 5 mg/kg infliximab (301). High levels of TNF-α have been reported at all stages of HIV infection, and correlate with high viral load, depletion of CD4^+^ T-cells and poor disease progression (302–304). The activation of the TNF pathway by TNF-α may facilitate HIV infection and immune activation through multiple pathways (305), and therefore a TNF-α inhibitor may be an effective therapeutic strategy. Nevertheless, only a limited number of clinical studies have reported on the safety and effectiveness of TNF-α inhibitors in PLWH. One review by Gallitano et al., summarized the use of TNF-α inhibitors in 27 published cases of patients with HIV/AIDS, and advises that TNF-α inhibitors may induce improvement in PLWH (306). However, anti-TNF-α therapy has a risk of inducing opportunistic infections and comorbid complications, such as infections by *Pneumocystis jirovecii*, invasive mycoses, and listeriosis (307), and its use in HIV infection must be carefully monitored. Further clinical studies are required to provide definitive data regarding the safety and effectiveness of TNF-α inhibitors in PLWH.

## Conclusion

In PLWH, HIV-related gut damage enhances gut permeability and promotes microbial translocation, which plays a critical role in the chronic immune activation seen in these patients. We have summarized information regarding multiple plasma biomarkers of gut damage and microbial translocation (**Table 1**), some of which have been widely used, to assess potential therapeutic strategies in PLWH. However, we cannot confidently conclude that the attenuation of microbial translocation would definitely lead to a decrease in immune activation. Other than microbial translocation, chronic immune activation in PLWH may also be driven by other factors, including persistence of the HIV reservoir, depletion of regulatory T-cells, and coinfection with other viruses or organisms (308–310). Furthermore, discordant results exist among different studies due to their heterogenous study design, and more robust and concordant evidence is required to validate the role that these biomarkers may potentially play in the management of PLWH in clinical settings or during interventions that target repair of the gut lining. Moreover, the validity of some biomarkers have not been confidently supported by histological evidence, which is deemed the gold standard to observe the intestinal barrier. Some factors may also interfere with the capability and the value of these biomarkers, such as food intake, genetic differences, medications. Numerous adjunctive therapeutic strategies, such as probiotic use, FMT, and antibiotic use have been investigated to optimize the condition of the gut; however, there remains a dearth of satisfactorily targeted therapeutic options to accurately and adequately repair the structural integrity of the leaky gut in a manner that would effectively prevent microbial translocation and the consequent systemic inflammation seen in PLWH. In the future, further targeted investigations are warranted to develop biomarkers and possible therapeutic strategies for the leaky gut present in PLWH, and should include collaborative efforts encompassing microbiology, clinical care, and pharmacology.

## Author contributions

JO, JY, and XZ contributed to revision of the literature and preparation of the initial draft of the manuscript. SI, VH, and HC critically reviewed and copy-edited the manuscript. J-PR and YC contributed to conception of the manuscript and provided critical revision. All authors have read and endorsed the submitted version of the manuscript. All authors contributed to the article.
